# A cluster-tree-based trusted routing algorithm using Grasshopper Optimization Algorithm (GOA) in Wireless Sensor Networks (WSNs)

**DOI:** 10.1371/journal.pone.0289173

**Published:** 2023-09-08

**Authors:** Mehdi Hosseinzadeh, Omed Hassan Ahmed, Jan Lansky, Stanislava Mildeova, Mohammad Sadegh Yousefpoor, Efat Yousefpoor, Joon Yoo, Lilia Tightiz, Amir Masoud Rahmani

**Affiliations:** 1 Institute of Research and Development, Duy Tan University, Da Nang, Vietnam; 2 School of Medicine and Pharmacy, Duy Tan University, Da Nang, Vietnam; 3 Department of Computer Science, University of Human Development, Sulaymaniyah, Iraq; 4 Department of Information Technology, University of Human Development, Sulaymaniyah, Iraq; 5 Department of Computer Science and Mathematics, Faculty of Economic Studies, University of Finance and Administration, Prague, Czech Republic; 6 Department of Computer Engineering, Dezful Branch, Islamic Azad University, Dezful, Iran; 7 School of Computing, Gachon University, Seongnam, Korea; 8 Future Technology Research Center, National Yunlin University of Science and Technology, Yunlin, Taiwan; TU Wien: Technische Universitat Wien, AUSTRIA

## Abstract

In wireless sensor networks (WSNs), existing routing protocols mainly consider energy efficiency or security separately. However, these protocols must be more comprehensive because many applications should guarantee security and energy efficiency, simultaneously. Due to the limited energy of sensor nodes, these protocols should make a trade-off between network lifetime and security. This paper proposes a cluster-tree-based trusted routing method using the grasshopper optimization algorithm (GOA) called CTTRG in WSNs. This routing scheme includes a distributed time-variant trust (TVT) model to analyze the behavior of sensor nodes according to three trust criteria, including the black hole, sink hole, and gray hole probability, the wormhole probability, and the flooding probability. Furthermore, CTTRG suggests a GOA-based trusted routing tree (GTRT) to construct secure and stable communication paths between sensor nodes and base station. To evaluate each GTRT, a multi-objective fitness function is designed based on three parameters, namely the distance between cluster heads and their parent node, the trust level, and the energy of cluster heads. The evaluation results prove that CTTRG has a suitable and successful performance in terms of the detection speed of malicious nodes, packet loss rate, and end-to-end delay.

## 1 Introduction

Wireless sensor network (WSN) is an important element for designing Internet of Things (IoT). It includes sensor nodes, which monitor the environment to gather data and send it to the base station [1, 2]. In a WSN-based IoT network, intelligent routing is a main and necessary phenomenon for improving the quality of service (QoS) [3, 4]. Furthermore, providing the energy needed for communications is a major challenge to decrease packet loss. In the routing process, it is necessary to prevent the rapid discharge of sensor nodes and unbalanced energy distribution in the network [5, 6]. Hence, it is essential to manage the energy consumed by nodes using intelligent machine learning techniques, metaheuristic algorithms, or other optimization strategies to make effective routing decisions and improve network performance [7, 8]. Many energy-efficient routing approaches are currently available in the literature for WSNs. However, they need to be enhanced for a WSN-based IoT environment [9, 10].

Today, IoT has improved universal access for deploying intelligent networks. A network edge provides intelligent services and computing for IoT devices [11, 12]. Additionally, this deployment improves user’s experience and presents efficient and flexible services when any unpleasant event occurs. Edge computing provides fast response and high-quality services because it utilizes an architecture close to end users [13, 14]. However, there are some security concerns, and security protocols must protect the network from the vulnerability of attacks (VoA). Existing techniques are mainly designed to infer intrusions in the network [15, 16]. However, determining secure and valid sensor nodes is a challenging issue in recent research. Moreover, attackers continuously change their locations to do their hostile activities around the network [17, 18]. Trust is an important component of cybersecurity. This component is responsible for determining the security level of sensor nodes during their interaction with each other. It actively identifies trusted nodes and prevents security risks caused by privacy violations, data manipulation or deletion, and other cybersecurity attacks [19, 20]. This illustrates the importance and necessity of trusted routing protocols.

Because of the special characteristics of sensor nodes, like small size, limited memory capacity, constrained energy source, and low computing power, energy consumption management is essential when designing a trust mechanism. Security and energy efficiency are two very important concepts in WSN-based IoT networks. However, they contradict each other. The deployment of these networks in vulnerable and unfriendly environments has led to their vulnerability to various attacks. Hence, existing routing protocols in WSNs need to implement powerful security mechanisms to secure the data transmission process. Although, the design of these mechanisms is associated with complex calculations that lead to high memory and energy consumption. Solving the challenges mentioned above is our main motivation for designing a trust-aware, energy-efficient, and lightweight routing algorithm. In this paper, a cluster-tree-based trusted routing approach using the grasshopper optimization algorithm (GOA) called CTTRG for WSNs is introduced. In addition to focusing on energy efficiency, the proposed scheme attempts to neutralize several routing attacks, especially black hole attack (BH), sinkhole attack (SH), wormhole attack (WH), gray hole attack (GH), and flooding attack (FA). To ensure security, CTTRG proposes a distributed time-variant trust (TVT) model to evaluate the trust of sensor nodes in the network. Also, to ensure energy efficiency, CTTRG uses a tree-cluster hierarchical topology to determine data transmission paths to the base station. In CTTRG, a technique to construct a GOA-based trusted inter-cluster routing tree (GTRT) is presented. BS is responsible for building this routing tree. In summary, the most important contributions of CTTRG are as follows:

- Designing a time-variant trust model based on three trust criteria, namely BH, SH, and GH probability, WH probability, and FA probability, and analyzing the behavior of sensor nodes when cooperating with each other.
- Adding a weight coefficient to the recommendations provided by the recommender nodes. The weight of each recommendation is determined according to the trust level of the recommender nodes and the difference between the recommended trust and the calculated direct trust.
- Constructing a trusted routing tree based on the grasshopper optimization algorithm to form stable and trusted communication paths between the cluster heads (CHs) and the base station (BS).
- Designing a multi-objective fitness function according to the distance between each CH and its parent node, the trust level of each CH, and its energy.

In the following, the organization of this paper is as follows: Section 2 exhibits the most important trusted routing methods in WSNs. In Section 3, the grasshopper optimization algorithm is presented in summary. Section 4 discusses network settings, energy model, and threat model in CTTRG. In Section 5, our method is introduced in detail. Section 6 describes the simulation and evaluation results. Finally, the conclusion of the paper is stated in Section 7.

## 2 Related works

In [21], a trust-aware and energy-efficient routing protocol called TBSEER has been proposed. It obtains the comprehensive trust of each sensor node with regard to three criteria, including adaptive direct trust, indirect trust, and energy. TBSEER counteracts BH, GH, SH, WH, and FA attacks. Furthermore, this approach presents an adaptive punishment structure to detect malicious nodes quickly. After obtaining direct trust based on the mentioned criteria, the sink is responsible for extracting indirect trust. In this case, sensor nodes conserve their energy because they do not perform repeated operations to calculate this parameter. Finally, CHs find secure paths between themselves and the sink node and consider their trust values in this process. This secure routing process protects the network against various attacks. Simulation results show that this method decreases energy consumption in the network. Also, this approach detects malicious nodes quickly and resists routing attacks.

In [22], a trust-aware and energy-efficient secure routing method called TESRP is introduced for WSNs. This scheme utilizes a decentralized trust structure to separate hostile nodes from honest nodes. Furthermore, TESRP employs a multi-facet routing mechanism to decide on routing paths according to trust value, remaining energy, and the number of hops. This strategy has two main advantages, namely secure data transfer and balanced energy consumption. The evaluation results prove that TESRP is better than other routing approaches in terms of consumed energy, throughput, and network longevity.

In [23], a lightweight and attack-resistant trust-based routing scheme called TSSRM is proposed for WSNs. This approach applies a secure path selection mechanism, which considers the trust value and QoS requirements. The goal of TSSRM is to counteract routing attacks and balance energy consumption in the data transmission procedure. TSSRM designs a secure routing strategy and measures the trust of nodes. In the trust evaluation process, this scheme analyzes the behavior of nodes based on their energy and movement. Then, this scheme discovers different paths between sensor nodes. In the secure route selection mechanism, TSSRM calculates the trust of the discovered paths to choose the most prominent routes. Finally, TSSRM merges QoS requirements and the trust value using the Semiring theory. Evaluations performed in this paper confirm the performance of this scheme.

In [24], a secure energy-efficient routing scheme called ECATS is suggested in WSNs with the mobile sink. It utilizes a fuzzy C-Means and an adaptive TDMA scheduling in the clustering process. Also, it introduces a path construction operation to comfort communications and render data packets to the sink node. ECAT presents a new encryption algorithm called Neural Elliptic Galois (NEG) to provide data security and privacy in the network. Additionally, ECATS finds cluster heads based on their consumed energy in the data aggregation operation. ECATS utilizes an ant lion optimization-based TDMA scheduling to enhance energy efficiency and network reliability at the same time. The evaluation results prove the successful performance of ECATS compared to other routing methods.

In [25], an energy balancing secure routing algorithm using the ant colony optimization called QEBSR is introduced for WSNs. QEBSR employs an event-oriented scenario in the data transfer procedure between nodes and BS. In addition, it utilizes an enhanced technique to calculate latency in the data transfer operation and extract the trust coefficient of nodes in the routing procedure. The ACO algorithm is responsible for searching paths using a max-min system. Eventually, the comparison of QEBSR with DEBR and EENC shows that this scheme has a successful performance.

In [26], the authors suggested a blockchain-based routing scheme in WSNs. In this approach, the blockchain technology designs a common storage capacity between sensor nodes to balance network traffic, lower interference, and enhance network security in the routing process. The authors have assumed that nodes sense events and produce a high volume of data. Hence, this data must be transferred in several packets. In this scheme, sensor nodes play the role of coins, and the transaction means the ownership exchanged between nodes and the sink node. Blockchain stores these transactions and shares the network state using a real-time manner. In the route selection procedure, this scheme introduces a cost function, which includes the load density and interference level of nodes. In addition, blockchain is responsible for protecting the discovered paths. Experiments prove that this scheme can be implemented in real-time systems.

In [27], the authors offered a cluster-based routing approach based on neuro-fuzzy rules called FBCFP to perform the routing operation in WSN-based IoT networks. FBCFP executes the network learning procedure based on the energy value, the distance from CHs to BS, the change in the cluster area, and the degree of CH. FBCFP learns the network environment using a convolutional neural network (CNN) and adjusts its initial weights using a fuzzy system. Furthermore, FBCFP employs a fuzzy system to create a strong clustering structure in the network. It considers similar sizes for clusters and utilizes the suitable rules for training the machine learning algorithm to optimize energy usage and QoS requirements in the WSN-based IoT network. According to the experiments performed in this paper, it can be found that FBCFP is well in terms of used energy, PDR, latency, and network longevity.

In [28], the authors suggested a secure routing protocol along with multiple-variant tuples. This scheme employs a symmetric cryptography strategy called Two-Fish (TF) method to detect and separate attackers on WSNs. Furthermore, this method includes an encryption mechanism and an authentication technique to provide security in the network. It utilizes Eligibility Weight Function (EWF) to find guard nodes. This function is protected using a symmetric cryptography technique. The evaluation results confirm that this scheme utilizes more monitoring nodes than other routing schemes. In addition, it deals with mobile attackers and improves packet delivery.

In [29], a cluster-based routing protocol is offered in a WSN-based IoT network. This scheme performs the routing process and the cluster head selection using two metaheuristic algorithms. The rider optimization algorithm (ROA) is employed to find cluster heads and improve QoS and reliability in the network. ROA uses a multi-objective fitness function, which depends on residual energy, distance, and delay. CHs are refreshed after certain iterations to guarantee load balancing in the network. Furthermore, the sailfish optimization algorithm (SFO) is used to select efficient and optimal routes between sensor nodes. This routing process considers several parameters namely throughput, remaining energy, and link quality. The evaluation results show that this scheme can improve execution time, energy consumption, network delay, throughput, packet delivery ratio, and network lifetime.

In [30], a trust-aware cluster-based routing algorithm is suggested in WSNs. This scheme compresses the sensed data in the data aggregation process to reduce overhead. On the other hand, this scheme implements various meta-heuristic algorithms such as artificial bee colony algorithm, ant colony optimization, differential evolution, firefly algorithm, and particle swarm optimization to validate the trust-aware routing process in WSN and make a trade-off between transmission distance, hop-count, number of transmitted messages, and trusted path. The base station has the responsibility to reconstruct the compressed data and check the trust of CHs. Moreover, CHs perform compressed sensing and trust-based data aggregation operations. These operations enhance security and limit overhead in each CH. In this scheme presents an objective function, which minimizes the distance traveled, number of hops, and number of messages and maximizes the trust related to the path.

In [31], a trust-aware routing method (TARM) is presented for WSN-based IoT networks. This scheme utilizes a mobile edge node to receive data from valid nodes. The edge node separates abnormal nodes from normal nodes based on a trust evaluation method. TARM performs the clustering process using a gray wolf optimizer and obtains trust values for each cluster. Then, the edge node receives data packets only from normal nodes through the corresponding cluster heads. TARM uses the artificial bee colony optimization to find the most suitable routes between valid nodes and the edge node. Simulations show that the trust evaluation mechanism proposed in TARM provides high security and has a high detection rate and high accuracy in detecting abnormal nodes. Also, this scheme conserves energy efficiently.

In [32], a trusted clustering protocol is proposed for WSNs. This scheme offers a trust model to detect untrusted nodes. This trust model considers two trust factors namely energy trust and data trust. In addition, this scheme utilizes stochastic fractal search optimization to do the clustering process. For maximizing network lifetime and improving network security, the clustering method proposes a fitness function to choose CHs from the trusted nodes. This function depends on the remaining energy, the number of nodes, the distance to the base station, and the dissipated energy. This clustering method can make load balancing among CHs. Evaluations show the superiority of this scheme in comparison with existing protocols.

In [33], a cluster-based routing approach is presented for heterogeneous WSNs. In the clustering process, K-means algorithm and cat swarm optimization are combined to obtain a new evolutionary approach called calf search optimization algorithm (K-CSOA), which is used to create clusters in the network. In the clustering process, K-CSOA presents a fitness function, which considers six factors, namely node degree, distance from cluster members to CHs, distance from CHs to BS, average and remaining energy, and balancing factor for clusters. The routing process uses ant colony optimization (ACO) to find the most suitable paths in the network. Simulations performed in this paper show the effectiveness of K-CSOA in terms of energy consumption and delay.

Table 1 compares our proposed scheme with the related works.

**Table 1:** Comparison of the related works.

Method	Publication year	Security mechanism	Routing technique	Energy efficiency	Strengths	Weakness
TBSEER [21]	2022	An adaptive trust mechanism based on a punishment factor	A trust-aware clustering routing protocol	✓	Considering the energy trust value in the trust evaluation mechanism, using an adaptive punishment factor to calculate the direct trust value, high accuracy, and high detection speed for identifying hostile nodes	Selecting CHs only based on their trust value
TESRP [22]	2016	A decentralized trust structure based on Beta probability density function	AODV protocol by considering a combination of nodes’ trust, remaining energy, and hop counts	✓	Detecting and isolating hostile nodes, scalability, considering energy parameter in the routing protocol	High routing overhead, high delay in the route discovery process
TSSRM [23]	2017	A trust evaluation mechanism based on Analytic hierarchy process (AHP)	An enhanced GPSR algorithm based on the trust degree and other QoS requirements	✓	Taking into account energy metric in the trust evaluation mechanism, executing many experiment scenarios	Falling into the local minimum, not considering a clustering process
ECATS [24]	2018	A NEG encryption algorithm and a fault node detection model	A clustering method based on fuzzy C-means and ant lion optimization	✓	Considering energy metric in the CH selection process	Not defining a routing process between CHs, not evaluating the resistance of this method against various attacks
QEBSR [25]	2019	A trust evaluation mechanism based on the packet drop rate and the packet generation rate	An ant colony optimization-based routing protocol	✓	Balanced energy consumption in the network, considering QoS requirements such as delay in the routing process	In some cases, the determination of weight vectors are not possible or very difficult, not evaluating the resistance of this scheme against various attacks, high time complexity
Lazrag et al. [26]	2019	Blockchain	Deciding on routing paths based on a cost function	×	Balancing traffic load, reducing interferences, and increasing security in the network	Not considering energy efficiency in the routing process
FBCFP [27]	2019	×	A cluster-based routing approach based on neuro-fuzzy rules	✓	Considering energy metric in the routing process, improving energy consumption in the network	High time complexity
Deebak and Al-Turjman [28]	2019	Designing an authentication mechanism and applying symmetric key approaches	A hybrid routing scheme based on OLSR and AOMDV	×	Ability to act as proactive and reactive routing protocol, detecting, preventing, and isolating hostile nodes	Not considering energy parameter in the routing process
Joshi and Raghuvanshi [29]	2021	×	A CH selection process based on the ROA algorithm and a routing process based on the SFO algorithm	✓	Making load balancing in the network, considering energy in the routing and clustering processes	High time complexity
Gilbert et al. [30]	2019	A beta-based trust evaluation system	A K-means-based clustering method and a routing protocol based on meta-heuristic algorithms	×	Low routing overhead, employing compressed sensing and data aggregation techniques, detecting abnormal nodes	Not considering the energy parameter in the routing process
TARM [31]	2022	A trust evaluation system	A GWO-based clustering method and a ABC-based routing algorithm	✓	High detection rate and high accuracy in detecting abnormal nodes	High time complexity, not evaluating the resistance of this scheme against various attacks
Hriez et al. [32]	2021	A trust mechanism based on energy trust and data trust	A trusted clustering process based on stochastic fractal search optimization	✓	Maximizing network lifetime, improving network security, making load balancing	High time complexity
K-CSOA [33]	2022	×	A cluster-based routing approach based on K-means algorithm and cat swarm optimization and a ACO-based routing process	✓	Low delay, low energy consumption	Not considering a security mechanism

## 3 Basic concepts

In recent decades, optimization algorithms inspired by nature have attracted the attention of researchers and academics. These algorithms have been used in engineering, computer science, and other fields to solve complex and real-world problems. In these algorithms, a set of solutions are generated and modified at each iteration to discover the optimal solution in the search space [34, 35]. Some nature-based algorithms include Particle Swarm Optimization (PSO) [36], Artificial Bee Colony (ABC) algorithm [37], Grey Wolf Optimizer (GWO) [38], Dragonfly Algorithm (DA) [39], and Grasshopper Optimization Algorithm (GOA) [40]. In 2017, Saremi et al. presented GOA, which simulates the food search behavior of grasshoppers in nature. Various studies have shown the use of this algorithm for solving many problems. For example, refer to [41–43]. In CTTRG, GOA is responsible for finding a secure and energy-efficient routing tree among cluster heads because the construction of a such routing tree among sensor nodes, especially in dense networks, is difficult and time-consuming. To solve this problem, GOA is chosen because it has been widely used in various fields, especially routing, and has proven its competence and effectiveness. In [44], extensive experiments have been conducted to evaluate GOA compared to other well-known algorithms such as PSO [36], Bat Algorithm (BA) [45], Flower Pollination Algorithm (FPA) [46], Cuckoo Search (CS) [47], Firefly Algorithm (FA) [48], Genetic Algorithms (GA) [49], Differential Evolution (DE) [50], and Gravitational Search Algorithm (GSA) [51]. These experiments have shown that GOA works very well and can be used to solve complex real-world problems because it can effectively balance exploration and exploitation and guide virtual grasshoppers towards the global optimum. In general, the most important advantages of GOA are high-quality exploration operations, avoidance of local optimum, and high convergence speed. The mathematical model presented in Eq 1 is used to model the behavior of grasshoppers in nature:

$$\begin{array}{c} X_{i}=S_{i}+G_{i}+A_{i} \end{array}$$ (1)

so that *i* is the index of grasshoppers, *X*_*i*_ indicates the position of grasshopper *i*, *S*_*i*_ shows the social interaction, *G*_*i*_ shows the gravity force, and *A*_*i*_ represents the direction of the wind. In order to create a random behavior, Eq 1 is written as *X*_*i*_ = *r*_1_*S*_*i*_ + *r*_2_*G*_*i*_ + *r*_3_*A*_*i*_ where *r*_1_, *r*_2_, and *r*_3_ are random coefficients in [0, 1].

$$\begin{array}{c} S_{i}=\sum_{\begin{array}{l} j=1\\ j\neq i \end{array}}^{N}s\left(d_{ij}\right)\hat{d_{ij}} \end{array}$$ (2)

so that *d*_*ij*_ is the distance from grasshopper *i* to grasshopper *j*. This distance is equal to *d*_*ij*_ = |*x*_*j*_ − *x*_*i*_|. Furthermore, $\hat{d_{ij}}=\frac{x_{j}-x_{i}}{d_{ij}}$ represents a unit vector drawn from grasshopper *i* to grasshopper *j*. *s* is used to express social forces. It is obtained through Eq 3.

$$\begin{array}{c} s(r)=fe^{\left(\frac{-r}{l}\right)}-e^{-r} \end{array}$$ (3)

so *f* and *l* are the attraction intensity and the attractive length, respectively. Change in these parameters causes different behaviors in grasshoppers. This social interaction can be defined as attraction and repulsion. Assume that the distance between two grasshoppers is between 0 and 15. If the distance is in [0, 2.079], the social interaction is repulsion. If the distance is 2.079, the grasshoppers are in the comfort area. Also, if the distance is in [2.079, 4], the social interaction is attraction.

*G* is computed according to Eq 4.

$$\begin{array}{c} G_{i}=-g\hat{e_{g}} \end{array}$$ (4)

where *g* displays the gravity constant, and $\hat{e_{g}}$ shows a unit vector.

*A* is obtained from Eq 5.

$$\begin{array}{c} A_{i}=u\hat{e_{w}} \end{array}$$ (5)

so that *u* is a fixed value and $\hat{e_{w}}$ represents a unit vector in the wind direction. After putting *S*, *G*, and *A* in Eqs 1 and 6 is obtained.

$$\begin{array}{c} X_{i}=\sum_{\begin{array}{l} j=1\\ j\neq i \end{array}}^{N}s(|x_{j}-x_{i}|)\frac{x_{j}-x_{i}}{d_{ij}}-g\hat{e_{g}}+u\hat{e_{w}} \end{array}$$ (6)

where $s(r)=fe^{\frac{-r}{l}}-e^{-r}$ and *N* represents the number of grasshoppers.

However, this equation cannot be used to solve optimization problems because it cannot do exploration and exploitation in the search space around a response. In this mathematical model, grasshoppers reach the comfort area speedily and they cannot be concentrated at a particular point. Therefore, this model is modified as Eq 7 to obtain the new positions of grasshoppers in each iteration.

$$\begin{array}{c} X_{i}^{d}=c(\sum_{\begin{array}{l} j=1\\ j\neq i \end{array}}^{N}c\frac{ub_{d}-lb_{d}}{2}s(x_{j}^{d}-x_{i}^{d})\frac{x_{j}-x_{i}}{d_{ij}})+\hat{T_{d}} \end{array}$$ (7)

where *ub*_*d*_ and *lb*_*d*_ indicate the upper and lower boundaries in the dimension *d*, respectively. $s(r)=fe^{\frac{-r}{l}}-e^{-r}$, $\hat{T_{d}}$ is the best solution in the search area, and *c* indicates the decreasing coefficient, which lowers the comfort area, repulsion area, and attraction area. Note that *S* in Eq 7 is almost similar to the component *S* in Eq 1. However, this equation does not regard gravity force (*G*) and assumes that the wind (*A*) moves always toward $\hat{T_{d}}$.

Eq 7 shows the next position of grasshoppers. It is dependent on their current position, the position of $\hat{T_{d}}$, and the positions of other grasshoppers. *c* is an adaptive factor and has been used twice in Eq 7. The leftmost *c* plays the role of inertial weight in PSO. It is used to lower the motion of grasshoppers around $\hat{T_{d}}$ and balance exploration and exploitation in this case. However, the second *c* reduces attraction, comfort, and repulsion areas. To balance exploration and exploitation, *c* must be reduced based on iterations. This mechanism strengthens exploitation by increasing iterations. *c* lowers the comfort area when increasing the number of iterations. *c* is obtained from Eq 8.

$$\begin{array}{c} c=c\;\text{max}\;-l\frac{c\;\text{max}\;-c\;\text{min}\;}{L} \end{array}$$ (8)

where *c*max = 1, *c*min = 0.00001, *l*, and *L* are the maximum threshold, the minimum threshold, the current iteration, and the maximum number of iterations, respectively.

## 4 System model

The system model is formed of three items: network settings, energy consumption mechanism, and threat model.

### 4.1 Network settings

**Fig 1:**
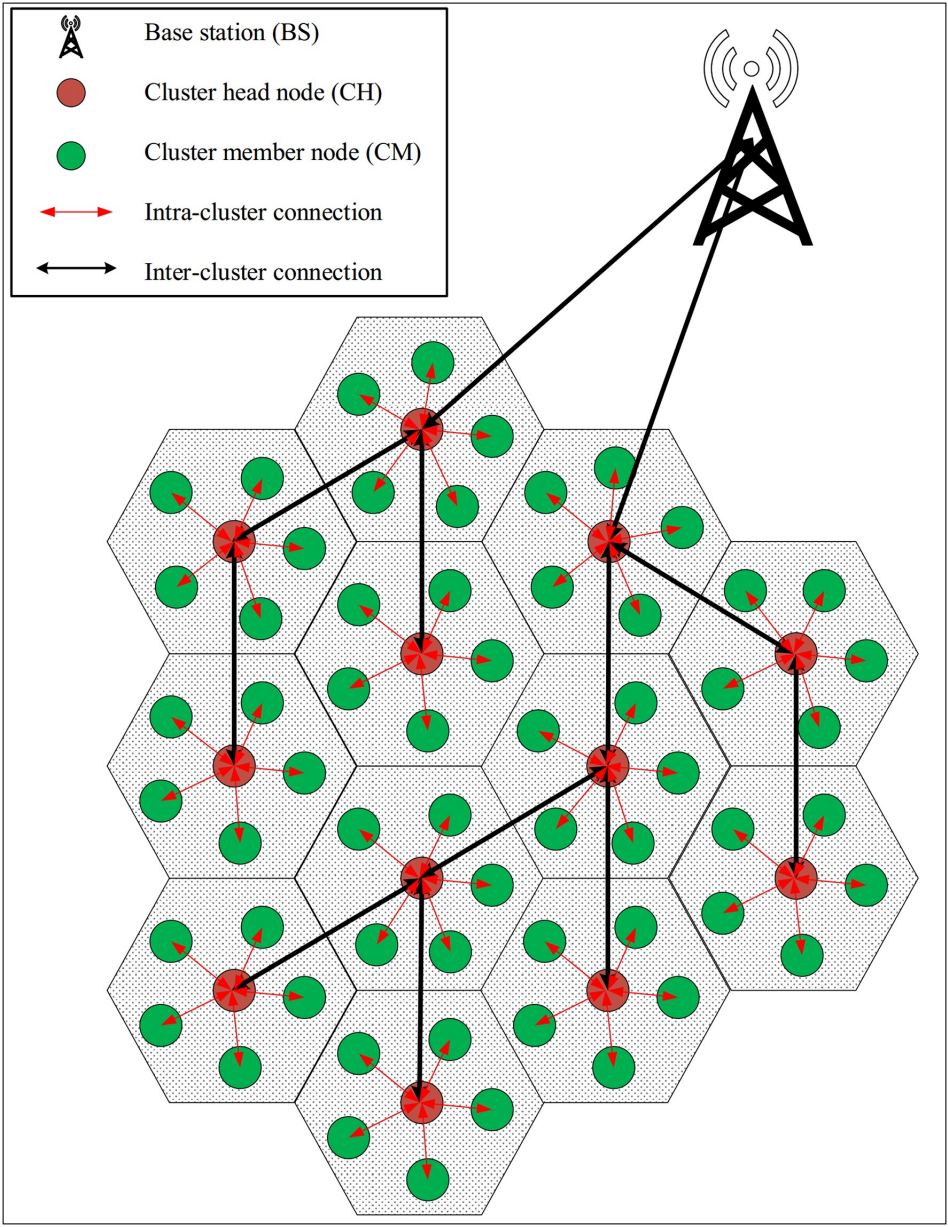
Network model in CTTRG.

In CTTRG, sensor nodes (i.e. *SN*_1_, *SN*_2_, …, *SN*_*i*_, …, *SN*_*N*_ so that *N* is the number of nodes) have been randomly arranged in the network environment. Fig 1 displays the network model. Additionally, the nodes are partitioned into multiple clusters using the LEACH algorithm, and CHs are selected from sensor nodes rotationally. The following assumptions are summarized for the network model used in CTTRG:

- Network nodes and the BS are static.
- BS utilizes an unlimited energy source.
- Network nodes are homogeneous, meaning that they use a similar energy source.
- Some equipment installed on sensor nodes are radio communication modules and positioning devices.
- The identifier of each *SN*_*i*_ is unique.

### 4.2 Energy consumption mechanism

In CTTRG, the energy model is defined in two modes, namely free space and multi-path. To transfer *k* bits to *SN*_*j*_, the energy used by *SN*_*i*_ is obtained from Eq 9.

$$\begin{array}{c} E_{TX}(k,d)=\{\begin{array}{c} E_{elec}\times k+E_{fs}\times k+d^{2},\;\;\;\;\;\;\;d<d_{0}\\ E_{elec}\times k+E_{mp}\times k+d^{4},\;\;\;\;\;\;\;d\geq d_{0} \end{array} \end{array}$$ (9)

Moreover, the energy used by *SN*_*j*_ to receive this packet is calculated according to Eq 10:

$$\begin{array}{c} E_{RX}(k,d)=E_{elec}\times k \end{array}$$ (10)

so that $d=\sqrt{{\left(x_{i}-x_{j}\right)}^{2}+{\left(y_{i}-y_{j}\right)}^{2}}$ indicates the distance between *SN*_*i*_ and *SN*_*j*_ with spatial coordinates (*x*_*i*_, *y*_*i*_) and (*x*_*j*_, *y*_*j*_), respectively. *E*_*elec*_ represents the energy used for the transmitter/receiver electrical equipment. Also, *E*_*fs*_ and *E*_*mp*_ are the energy needed by an amplifier in the free space model and the multi-path model, respectively. *d*_0_ expresses the transfer distance threshold so that $d_{0}=\sqrt{\frac{E_{fs}}{E_{mp}}}$.

### 4.3 Attack model

In WSNs, there is a need to prevent or reduce security risks caused by dynamic topology, deploying in dangerous environments, lack of a central controller, and wireless links. Trust is an important component in cybersecurity. It determines the trust level of each node when interacting with other sensor nodes [52, 53]. In fact, a security system seeks to actively identify reliable nodes and reduce security risks because these risks may violate privacy, manipulate or delete data, and provide a bed for other cybersecurity attacks. This shows the importance of a trusted routing protocol [54, 55]. In this paper, CTTRG deals with routing attacks, especially black hole (BH), sinkhole (SH), wormhole (WH), gray hole (GH), and flooding attack (FA).

- The BH node communicates with other nodes and creates fake paths in the network. The goal of this communication is to prevent data packets from being delivered to the destination and delete all the packets. To build fake routes, the BH node is waiting to receive route requests (RREQs) from other network nodes. As soon as the request is received, the BH node quickly responds to the requesting node. Note that these routes are fake, and in fact, there is not any path to the desired node [56, 57]. Additionally, to increase the attractiveness of these fake routes and absorb network traffic, the BH node adjusts the parameters associated with these paths such as delay and hops in the best possible case.
- The SH node is similar to the BH node, except that the SH node is aware of the position of the sink node and tries to attract all traffic toward the sink. Then, it prevents the packets from being sent to the sink. The attack is more dangerous than BH.
- A GH node is similar to BH, except that GH is smarter. GH does not eliminate all data packets, but focuses on a particular type of packets or on a specific node and removes all packets sent to that node, in other cases, it shows a normal behavior [58, 59]. As a result, it is difficult to identify GH.
- WH attack will be carried out by two attacker nodes. These two nodes create a tunnel between themselves and encourage other nodes to use this tunnel for sending their data packets. They make this tunnel very attractive in terms of routing parameters to attract the network traffic. The attack provides a suitable bed for tracking the communications of transmitter nodes, copying data packets, manipulating the packets, or removing them.
- The FA node targets a specific node and sends a large number of fake route requests to it. Since the target node processes these requests and stores some information, its energy level is greatly reduced, and its memory overflows. Hence, the target node cannot respond to the real requests of legal nodes. Because of the constrained energy of sensor nodes, this attack causes serious damage to the network [60, 61].

## 5 The proposed method

**Fig 2:**
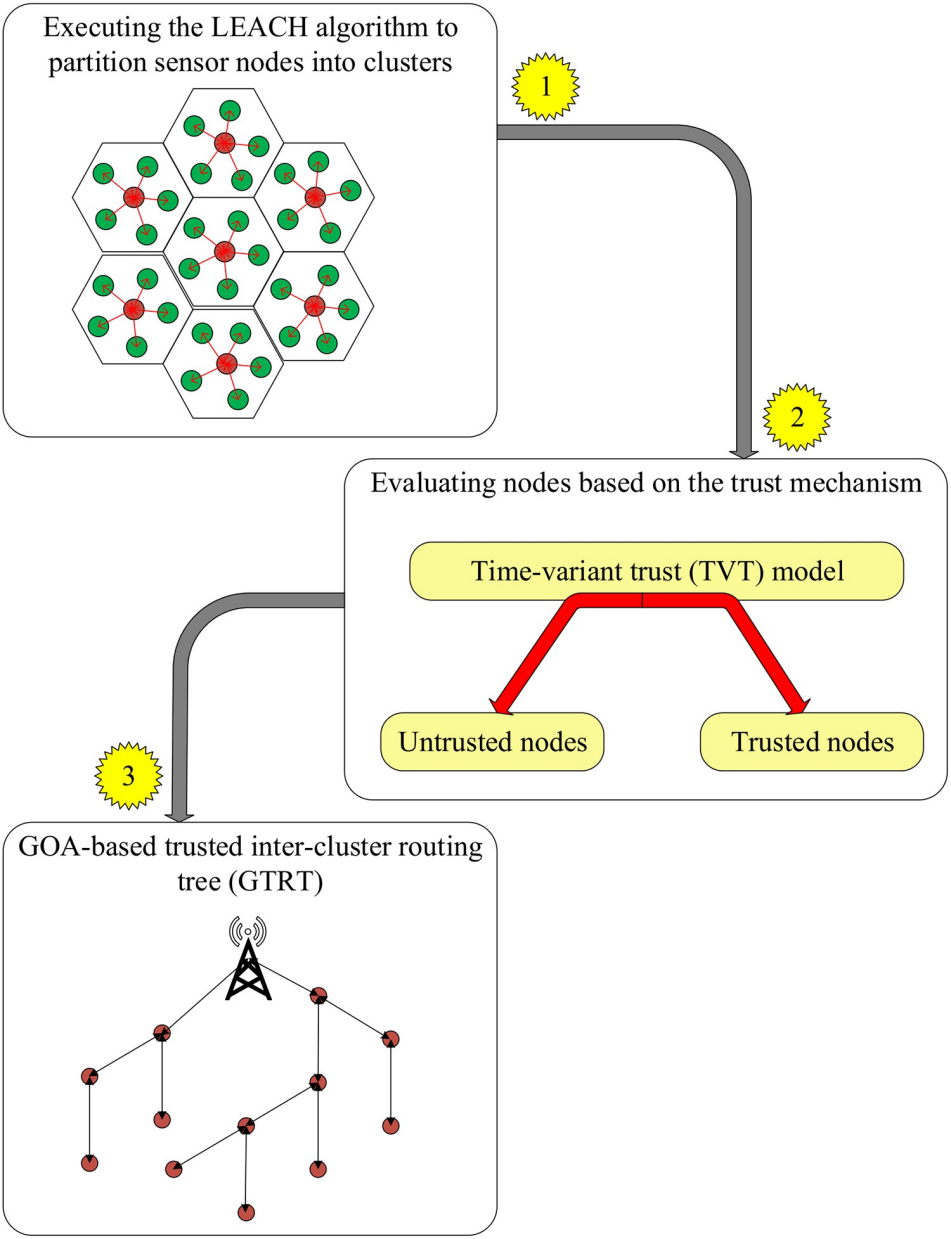
Diagram of CTTRG.

In this section, the cluster-tree-based trusted routing method using the GOA algorithm (CTTRG) will be introduced for wireless sensor networks. This method includes two main mechanisms: the time-variant trust (TVT) model and the GOA-based trusted inter-cluster routing tree (GTRT). A diagram of proposed method is presented in Fig 2.

### 5.1 Time-variant trust (TVT) model

In a conventional trust model, the trust of nodes is periodically refreshed, but the trust value is constant in each period. Whereas, this is not true, and trust is a time-variant variable and has no fixed value in each period. Therefore, if a time-variant weight coefficient is considered for trust parameters, it can provide a more accurate estimation of the trust value. In CTTRG, a decentralized time-variant trust model is proposed to get the trust value of nodes. TVT contains three components: time-variant direct trust (TVDT), recommended trust (RT), and time-variant final trust (TVFT).

#### 5.1.1 TVDT component

In CTTRG, the TVDT component includes an initial value and a dynamic coefficient. The initial value is dependent on the three trust criteria, namely the BH, SH, and GH probability (*p*_*SBG*_), the WH probability (*p*_*WH*_), and the FA probability (*p*_*FA*_). These three criteria are defined based on the analysis of the behavior of sensor nodes when interacting with each other. Now, suppose that *SN*_*i*_ attempts to obtain an accurate estimation of the trust value corresponding to *SN*_*j*_. To achieve this goal, *SN*_*i*_ interacts directly with *SN*_*j*_ to acquire three criteria $p_{SBG}^{j}$, $p_{WH}^{j}$, and $p_{FA}^{j}$.

-
$p_{SBG}^{j}$ : This criterion examines the possibility that *SN*_*j*_ is a SH node, a BH node, or a GH node. These three attacks are very similar to each other and have little difference, which was discussed in Section 4.3. The most important feature of the SH, BH, and GH nodes is that they have very low packet reception and sending rates and delete all (or more) data packets. Therefore, $p_{SBG}^{j}$ is obtained according to Eq 11.
  $$\begin{array}{c} p_{SBG}^{j}=\lambda (1-\frac{PK_{j}^{received}}{PK_{j}^{total-receiving}})+(1-\lambda )(1-\frac{PK_{j}^{sent}}{PK_{j}^{total-sending}}) \end{array}$$ (11)
  where $PK_{j}^{received}$ indicates the number of packets received by *SN*_*j*_ and $PK_{j}^{total-receiving}$ is the total number of packets that should be received by *SN*_*j*_. Moreover, $PK_{j}^{sent}$ expresses the number of packets sent by *SN*_*j*_ and $PK_{j}^{total-sending}$ indicates the total number of packets, which should be sent by *SN*_*j*_. λ is also a fixed number adjusted in [0, 1]. λ expresses the weight associated with the packet reception rate and determines the relative importance of the packet reception rate and the packet sending rate. This weight can be adjusted based on the requirements of the application.
-
$p_{WH}^{j}$ : This criterion examines the possibility that *SN*_*j*_ is a WH node. The most important feature of WH nodes is that they tend to form various paths and absorb the traffic of the surrounding nodes. This tendency to absorb network traffic causes congestion in the WH nodes and consequently they experience a very long queuing delay. The second feature of these nodes is low package reception rate because they eliminate many received packets. Another feature of WH nodes is that they copy some data packets and relay the duplicated packets on the network. Therefore, they experience high redundancy rate. Finally, $p_{WH}^{j}$ is defined in Eq 12:
  $$\begin{array}{c} p_{WH}^{j}=\Psi_{1}(\frac{T_{j}^{Q}}{\max_{SN_{k}\in N_{i}}\;\{T_{k}^{Q}\}})+\Psi_{2}(1-\frac{PK_{j}^{received}}{PK_{j}^{total}})+\Psi_{3}(\frac{DPK_{j}}{NPK_{j}+DPK_{j}}) \end{array}$$ (12)
  where $T_{j}^{Q}$ indicates a queuing delay of *SN*_*j*_. This parameter is inserted into hello packets. *N*_*i*_ expresses the set of neighbors of *SN*_*i*_. Finally, *NPK*_*j*_ and *DPK*_*j*_ describe the number of new and duplicate packets received from *SN*_*j*_, respectively. In addition, Ψ_1_ is the weight coefficient related to the delay parameter, Ψ_2_ is the weight coefficient related to the packet reception rate, and Ψ_3_ is the weight coefficient related to the redundancy rate such that Ψ_1_, Ψ_2_, and Ψ_3_ are fixed numbers in [0, 1] and $\sum_{i=1}^{3}\Psi_{i}=1$. These weights show the importance of these factors and can be set in accordance with the requirements of the application.
-
$p_{FA}^{j}$ : This criterion examines the probability that *SN*_*j*_ is a FA node. The most important feature of FA nodes is high energy consumption, and the second feature is high route request sending rate. Another feature of these nodes is a large number of duplicate packets. According to the mentioned points above, Eq 13 calculates $p_{FA}^{j}$.
  $$\begin{array}{c} p_{FA}^{j}=\ell_{1}\frac{\left(\frac{E_{j}^{res,t-1}-E_{j}^{res,t}}{E_{ini}}\right)}{\Delta t}+\ell_{2}(\frac{\frac{PK_{j}^{sent}}{\max_{k\in N_{j}\;\;and\;\;SN_{j}}\;\{PK_{k}^{sent}\}}}{\Delta t})+\ell_{3}(\frac{DPK_{j}}{NPK_{j}+DPK_{j}}) \end{array}$$ (13)
  where *ℓ*_1_ is the weight coefficient related to the energy factor, *ℓ*_2_ is the weight coefficient related to the packet sending rate, and *ℓ*_3_ is the weight coefficient related to the redundancy rate such that *ℓ*_1_, *ℓ*_2_, and *ℓ*_3_ are fixed numbers in [0, 1] and $\sum_{i=1}^{3}\ell_{i}=1$. These weights show the importance of these factors and can be set in accordance with the requirements of the application. $E_{j}^{res,t}$ and $E_{j}^{res,t-1}$ represent the remaining energy of *SN*_*j*_ in two moments *t* and *t* − 1, respectively. *E*_*ini*_ is the initial energy of sensor nodes. $E_{j}^{res,t}$ is defined in Eq 14.
  $$\begin{array}{c} E_{j}^{res,t}=E_{ini}-EC_{j}^{t} \end{array}$$ (14)
  where $EC_{j}^{t}$ expresses the energy consumption of *SN*_*j*_ at the moment *t*. It is obtained from Eq 15 according to the energy model stated in Section 4.2.
  $$\begin{array}{c} EC_{j}=\sum_{x=1}^{n_{EC}}(E_{tx}^{j}+E_{rx}^{j}) \end{array}$$ (15)
  where $E_{tx}^{j}$, $E_{rx}^{j}$, and *n*_*EC*_ express the energy needed to send the packets, the energy needed to receive the packets, and the number of data transfer operations performed by *SN*_*j*_, respectively.

Finally, the initial value of the TVDT component in [*t* − 1, *t*] (i.e. *TVDT*_*ij*_(*t* − 1)) is defined in Eq 16:

$$\begin{array}{c} TVDT_{ij}(t-1)=1-\max\{p_{SBG}^{j},p_{WH}^{j},p_{FA}^{j}\} \end{array}$$ (16)

Now, *TVDT*_*ij*_(*t*) will be calculated by Eq 17.

$$\begin{array}{c} TVDT_{ij}(t)=TVDT_{ij}(t-1)e^{-\rho t},\;\;\;[t-1,t] \end{array}$$ (17)

so that *e*^−*ρt*^ is the time-variant dynamic coefficient. In this coefficient, *ρ* is equal to the standard value of *TVDT*_*ij*_(*t* − 1), which is obtained from Eq 18.

$$\begin{array}{c} \rho =\frac{TVDT_{ij}(t-1)-\mu_{TVDT}}{\sigma_{TVDT}} \end{array}$$ (18)

where *μ*_*TVDT*_ and *σ*_*TVDT*_ are the mean and standard deviation of *TVDT*_*ij*_(*t*) calculated by Eqs 19 and 20.

$$\begin{array}{c} \mu_{TVDT}=E(TVDT_{ij})=\int_{t=0}^{t-1}tTVDT_{ij}(t)dt \end{array}$$ (19)

$$\begin{array}{c} \sigma_{TVDT}=\sqrt{E(TVDT_{ij}^{2})-{\left(E(TVDT_{ij})\right)}^{2}} \end{array}$$ (20)

#### 5.1.2 The RT component

In this section, the recommended trust component in the TVT model will be introduced. RT represents that *SN*_*i*_ not only relies on its interactions to calculate the trust of *SN*_*j*_, but also uses the trust values recommended by the recommended nodes (*RN*_*k*_). In TVT, *RN*_*k*_ is a common node between *SN*_*i*_ and *SN*_*j*_, and *R* = {*RN*_1_, *RN*_2_, …, *RN*_*k*_, …, *RN*_|*R*|_} is a set that contains all *RN*_*k*_ nodes. In TVT, *SN*_*i*_ considers a weight coefficient *CT*_*ik*_(*t* − 1) for accepting the trust recommended by each *RN*_*k*_. This coefficient expresses the importance of the recommendation provided by *RN*_*k*_. It includes two criteria and is obtained according to Eq 21:

- **Initial trust of **SN**_**i**_ relative to **RN**_**k**_ (**TVDT**_**ik**_**(t − 1)**):** According to this criterion, *SN*_*i*_ does not consider the recommendation provided by an unreliable *RN*_*k*_.
- **The difference between the trust recommended by **RN**_**k**_ and the trust calculated by **SN**_**i**_:** According to this criterion, *SN*_*i*_ prefers the *RN*_*k*_ nodes that the TVDT calculated by them is closer to TVDT calculated by *SN*_*i*_.
$$\begin{array}{c} CT_{ik}(t-1)=TVDT_{ik}(t-1)(1-\frac{\left|TVDT_{ij}(t-1)-TVDT_{kj}(t-1)\right|}{\max_{RN_{k}\in R}\;\{TVDT_{kj}(t-1)\}}) \end{array}$$ (21)

According to the above criteria, *RT*_*ij*_ is calculated according to Eq 22.

$$\begin{array}{c} RT_{ij}=\frac{1}{\left|R\right|}\sum_{k\in R}^{\left|R\right|}(CT_{ik}(t-1)\cdot TVDT_{kj}(t-1)) \end{array}$$ (22)

so that *TVDT*_*kj*_(*t* − 1) is the initial trust value of *RN*_*k*_ relative to *SN*_*j*_, and |*R*| is the number of members of *R* = {*RN*_1_, *RN*_2_, …, *RN*_*k*_, …, *RN*_|*R*|_}.

#### 5.1.3 TVFT component

Now, given that TVDT is a time-variant function. Therefore, TVFT is also defined as a time-variant trust function provided in Eq 23.

$$\begin{array}{c} TVFT_{ij}^{t}=\alpha TVDT_{ij}(t)+(1-\alpha )RT_{ij} \end{array}$$ (23)

so that *α* ∈ [0, 1] is a regulatory coefficient.

Algorithm 1 describes how to calculate the trust values of sensor nodes. The time complexity of this algorithm is calculated based on the following steps:

- Lines 1 and 2 of Algorithm 1 includes two nested *For* loops so that each loop is repeated *N* times.
- There is an *IF* command inside these nesting loops. It includes the following commands:
  - Lines 4 to 9 consist of 6 commands with fixed run times *r*_1_, *r*_2_, *r*_3_, *r*_4_, *r*_5_, and *r*_6_, respectively.
  - Line 10 contains a *For* loop, which is repeated |*R*| times and has four commands (lines 11 to 14) with fixed run times *r*_7_, *r*_8_, *r*_9_, and *r*_10_, respectively.
    $$\begin{array}{c} T_{For}(N)=\left|R\right|(r_{7}+r_{8}+r_{9}+r_{10}) \end{array}$$ (24)
    Suppose that there is a fixed number such as *r* so that *r* > *r*_7_+*r*_8_+*r*_9_+*r*_10_. In this case, Eq 24 is rewritten to obtain Eq 25:
    $$\begin{array}{c} T_{For}(N)=\left|R\right|(r_{7}+r_{8}+r_{9}+r_{10})<\left|R\right|(r) \end{array}$$ (25)
  - Lines 16 and 17 are two commands with fixed execution times, *r*_11_ and *r*_12_, respectively.
  Hence, the overall execution time of this *IF* is obtained from Eq 26:
  $$\begin{array}{c} T_{IF}(N)=r_{1}+r_{2}+r_{3}+r_{4}+r_{5}+r_{6}+\left|R\right|(r)+r_{11}+r_{12} \end{array}$$ (26)
  If a fixed number like *p* is considered:
  $$\begin{array}{c} T_{IF}(N)=r_{1}+r_{2}+r_{3}+r_{4}+r_{5}+r_{6}+\left|R\right|(r)+r_{11}+r_{12}<p\left|R\right| \end{array}$$ (27)

According to the above, the time complexity of Algorithm 1 is calculated based on Eq 28:

$$\begin{array}{c} T(N)=N^{2}(T_{IF}(N))=N^{2}\left|R\right| \end{array}$$ (28)

so that *N* indicates the number of sensor nodes and |*R*| represents the number of recommender nodes.

**Algorithm 1** Time variant trust model (TVT model)

**Input:** *SN*_1_, *SN*_2_, …, *SN*_*i*_, …, *SN*_*N*_: Sensor nodes in the network

**Output:** $TVFT_{ij}^{t}$ : Time variant final trust of *SN*_*j*_ estimated by *SN*_*i*_.

  **Begin**

1: **for** *i* = 1 to *N*

2:  **for** *j* = 1 to *N* **do**

3:   **if** *i* ≠ *j* **AND** *SN*_*i*_ and *SN*_*j*_ are neighbors **then**

4:    **SN**_**i**_: Calculate $p_{SBG}^{j}$ using Eq 11;

5:    **SN**_**i**_: Evaluate $p_{WH}^{j}$ using Eq 12;

6:    **SN**_**i**_: Obtain $p_{FA}^{j}$ from Eq 13;

7:    **SN**_**i**_: Calculate *TVDT*_*ij*_(*t* − 1) using Eq 16;

8:    **SN**_**i**_: Get *ρ* in accordance with Eq 18;

9:    **SN**_**i**_: Achieve *TVDT*_*ij*_(*t*) based on Eq 17;

10:    **for** *k* = 1 to |*R*| **do**

11:     **SN**_**i**_: Assess *TVDT*_*ik*_(*t* − 1) according to Eq 16;

12:     **RN**_**k**_: Compute *TVDT*_*kj*_(*t* − 1) based on Eq 16;

13:     **SN**_**i**_: Obtain the difference between *TVDT*_*ij*_(*t* − 1) and *TVDT*_*kj*_(*t* − 1);

14:     **SN**_**i**_: Calculate the weight coefficient *CT*_*ik*_(*t* − 1) according to Eq 21;

15:    **end for**

16:    **SN**_**i**_: Compute *RT*_*ij*_ by Eq 22;

17:    **SN**_**i**_: Obtain $TVFT_{ij}^{t}$ from Eq 23;

18:   **end if**

19:  **end for**

20: **end for**

  **End**

### 5.2 GOA-based trusted inter-cluster routing tree (GTRT)

In CTTRG, a GTRT tree is formed on the network to establish reliable connections between CHs and BS. BS uses the GOA algorithm to build a GTRT tree. It acquires information related to each CH node, for example, the distance to the BS, the trust level, and energy through the periodic exchange of hello messages. Furthermore, it puts all CHs in a set such as *TR* = {*CH*_1_, *CH*_2_, …, *CH*_*q*_, …, *CH*_*Q*_} (so that *Q* is the number of CHs in the network). In the routing tree construction issue, each grasshopper acts as a GTRT tree and specifies the routing path between each CH and BS. The following steps are executed to find the best GTRT tree:

- **Population formation:** Each grasshopper plays the role of a GTRT tree and specifies the arrangement of CHs in the tree. This grasshopper is shown as an array with *Q* elements so that each element of this array represents the spatial coordinates of CH. In the population formation process, a CH is randomly chosen from the *TR* set, and its spatial coordinates are inserted into the relevant element of the array.
- **GTRT tree corresponding to each grasshopper:** This step contains four stages to extract a GTRT tree from a grasshopper:
  - **Stage 1:** In all grasshoppers, BS corresponds to the root of the GTRT tree.
  - **Stage 2:** In each grasshopper, the first and second elements of the array are the left and right children of BS namely *LP* and *RP*, respectively.
  - **Stage 3:** Note that GTRT is a binary tree, and at each level of this tree, the leftmost parent first identifies its left and right children based on the relevant array. For example, the third and fourth elements of the array are known as left and right children of *LP*, and the fifth and sixth elements of the array are known as left and right children of *RP*.
  - **Stage 4:** Stage 3 is repeated to join all CHs to the relevant tree.
- **Evaluation:** In the GTRT tree construction algorithm, a multi-objective fitness function is considered to evaluate GTRT trees. Then, the positions of grasshoppers will be updated based on this fitness function in each iteration. The purpose of this update process is to change the positions of cluster heads in the routing tree and build the most suitable GTRT tree. To achieve this goal, the GTRT tree construction algorithm considers a multi-objective strategy so that the GTRT tree is built based on three factors, i.e. the distance between CHs and their parent node, the remaining energy of the cluster heads, and their trust level. In this regard, GTRT trees are evaluated in accordance with the fitness function in Eq 29.
  $$\begin{array}{c} F_{fitness}=\beta f_{1}+(1-\beta )f_{2} \end{array}$$ (29)
  so that *β* is a fixed number in [0, 1] that determines the effect of *f*_1_ and *f*_2_ on *F*_*fitness*_. After this evaluation, BS identifies the best response ($\hat{T_{d}}$) in the population.
  In the GTRT problem, the BS is looking for a tree in which the distance between each CH to its parent is short. The reason behind the selection of this factor is that in the data transmission process between a cluster head node and the base station, if the distance between each cluster head and its parent node is the shortest, this cluster head will transmit data packets to its parent node in the GTRT tree at a high speed (less delay). As a result, it will consume less energy in the data transfer process. Hence, *f*_1_ focuses on the distance of each CH to its parent and is calculated through Eq 30.
  $$\begin{array}{c} f_{1}=\frac{1}{\sum_{i=1}^{Q}d(CH_{i},Parent_{i})} \end{array}$$ (30)
  where $d(CH_{i},Parent_{i})=\sqrt{{\left(x_{i}-x_{p}\right)}^{2}+{\left(y_{i}-y_{p}\right)}^{2}}$. Also, (*x*_*i*_, *y*_*i*_) and (*x*_*p*_, *y*_*p*_) express the coordinates of *CH*_*i*_ and its parent (*Parent*_*i*_), respectively.
  On the other hand, energy is a very effective factor on network performance because the energy of cluster heads is dropped after performing several data transmission processes. Therefore, if the cluster head nodes with less energy are placed in the higher levels of the GTRT tree, their energy will be depleted faster because the nodes placed in the higher levels of the GTRT tree must transmit more data packets, as a result, their energy consumption will be higher. Note that in addition to sending the data related to their cluster members, these nodes must also transmit the data received from the cluster heads in their subtree to the base station. Also, BS considers the trust level of CHs in the fitness function to build the most secure GTRT tree among the cluster head nodes. The meaning of the most secure routing tree is that nodes with higher trust level are placed in the higher levels of the GTRT tree because as mentioned above, these nodes have more responsibilities and their security is more important than the nodes in the lower levels of the GTRT tree. Hence, the BS is looking for a tree that puts the more secure and high-energy CHs at the higher level of GTRT. As a result, *f*_2_ focuses on the order of CHs in GTRT based on their energy and trust through Eq 31.
  $$\begin{array}{c} f_{2}=\sum_{D=1}^{\left\lfloor \text{log}\;\;Q\right\rfloor }\frac{1}{D}\sum_{x=1}^{2^{D}}(\partial (\frac{E_{res,t}^{x}-E_{\;\text{min}\;}}{E_{ini}-E_{\;\text{min}\;}})+(1-\partial )(\frac{TVFT_{x}(t)-\min_{CH_{k}\in TR}\;\{TVFT_{k}(t)\}}{\max_{CH_{k}\in TR}\;\{TVFT_{k}(t)\}-\min_{CH_{k}\in TR}\;\{TVFT_{k}(t)\}})) \end{array}$$ (31)
  where $E_{res,t}^{x}$ describes the remaining energy of *CH*_*x*_, *E*_min_ = 15%*E*_*ini*_ is the minimum energy threshold, and *E*_*ini*_ indicates the primary energy of the network nodes. Furthermore, *TVFT*_*x*_(*t*) is the trust of *CH*_*x*_, and *D* indicates the tree depth, and ∂ is a fixed number in [0, 1].
- **End condition:** This stage specifies the stopping condition of the GTRT algorithm, so that the GTRT algorithm is run on 300 iterations, and the optimized GTRT is determined at the final iteration. Finally, BS informs the status of CHs in GTRT by sending a GTRT message that includes the arrangement of CHs in the routing tree.
- **Grasshopper updating operation:** The position of CHs in the relevant grasshopper will be refreshed using Eq 7.

Algorithm 2 explains how to build a GTRT tree. Time complexity of Algorithm 2 is obtained based on the following steps:

- Line 1 contains a command with a constant execution time *c*_1_.
- Line 2 is a *While* loop and emphasizes that Algorithm 2 is repeated throughout the simulation period (i.e. *t*_*sim*_).
- In Line 3, there is an *IF* condition inside this *While* loop.
  - Inside this *IF* command, there is a *For* loop (lines 4–7). This loop is repeated *Q* times and includes two commands (Lines 5 and 6) with fixed run times *c*_2_ and *c*_3_.
    $$\begin{array}{c} T_{For}(N)=Q(c_{2}+c_{3}) \end{array}$$ (32)
    If we consider a fixed number such as *c* so that *c* > *c*_2_+*c*_3_. In this case, Eq 32 is rewritten to obtain Eq 33:
    $$\begin{array}{c} T_{For}(N)=Q(c_{2}+c_{3})<Q(c) \end{array}$$ (33)
  Therefore, the overall execution time of this *IF* is obtained from Eq 34:
  $$\begin{array}{c} T_{IF}(N)=Q(c) \end{array}$$ (34)
- In line 9, there is an *IF* command that includes the following commands:
  - Lines 10, 11 and 12 have three commands with fixed run times *c*_4_, *c*_5_, and *c*_6_.
  - Time complexity of Line 13 is equal to *O*(*Q*).
  - Time complexity of Line 14 is equal to *O*(*Q*).
  - Time complexity of Line 15 depends on the fitness function presented in Eq 29. Its time complexity is equal to *O*(*Q*).
  - Line 16 is executed at a fixed time *c*_6_.
  Therefore, the run time of this *IF* is obtained from Eq 35:
  $$\begin{array}{c} T_{IF}(N)=c_{4}+c_{5}+c_{6}+3O(Q)+c_{6} \end{array}$$ (35)
  There is a fixed number such as *h*, which meets the following condition:
  $$\begin{array}{c} T_{IF}(N)=c_{4}+c_{5}+c_{6}+3Q+c_{6}<hQ \end{array}$$ (36)
- In Line 17, a *While* loop is repeated 300 times (It is the end condition of the GOA algorithm. Generally, it is displayed as *K*).
  - Lines 18 and 19 contain two commands with fixed run times *a*_1_ and *a*_2_, respectively.
  - Line 20 is dependent on the number of grasshoppers (for example, *PG*).
  - Time complexity of Line 21 is equal to *O*(*Q*).
  - Time complexity of Line 22 depends on the fitness function and is equal to *O*(*Q*).
  - Time complexity of Line 23 is determined based on the number of grasshoppers.
  Therefore, the run time of this *While* is obtained from Eq 37:
  $$\begin{array}{c} T_{While}(N)=K(a_{1}+a_{2}+2PG+2Q) \end{array}$$ (37)
  IF *PG* < *Q* and there is a fixed number such as *a*, the run time of this *While* is calculated based on Eq 38:
  $$\begin{array}{c} T_{While}(N)=K(a_{1}+a_{2}+2PG+2Q)<a(KQ) \end{array}$$ (38)
- Line 25 has a fixed runtime.

According to the points mentioned above, the time complexity of Algorithm 2 is *O*(*KQ*), so that *K* is equal to the end condition and *Q* is the number of cluster heads.

**Algorithm 2** GOA-based trusted routing tree (GTRT)

**Input:** *TR* = {*CH*_1_, *CH*_2_, …, *CH*_*q*_, …, *CH*_*Q*_}: Cluster head nodes

 BS: Base station

 *t*_*sim*_: Simulation time

 *t*_*hello*_: Hello message time period

 *t*_*couter*_: Timer

**Output:** The best GTRT

  **Begin**

1: *t*_*counter*_ = 0;

2: **while** *t*_*counter*_ ≤ *t*_*sim*_ **do**

3:  **if** $t_{counter}\;\;\;\text{mod}\;\;\;t_{hello}=0$ **then**

4:  **for** *q* = 1 to *Q* **do**

5:    **CH**_**q**_: Forward a Hello packet to the base station;

6:    **BS:** Save the position, the trust amount, and the remaining energy of *CH*_*q*_ in its storage space;

7:   **end for**

8:  **end if**

9:   **if** CHs change in the network **then**

10:   **BS:** Determine the number of grasshoppers in the GTRT algorithm;

11:   **BS:** Specify *c*max, *c*min, and the stop condition in the GTRT algorithm;

12:   **BS:** Consider an array with *Q* elements corresponding to each grasshopper;

13:   **BS:** Fulfill each element of grasshoppers with selecting CHs from the *TR* set randomly;

14:   **BS:** Extract GTRT trees from grasshoppers;

15:   **BS:** Evaluate each GTRT tree based on fitness function presented in Eq 29;

16:   **BS:** Determine the best grasshopper and set it as $\hat{T_{d}}$;

17:   **while** Stop condition is not met **do**

18:    BS: Update the coefficient *c* using Eq 8;

19:    **BS:** Normalize the distance between grasshopper in [1, 4];

20:    **BS:** Update grasshoppers based on Eq 7;

21:    **BS:** Extract GTRT trees from grasshoppers;

22:    **BS:** Evaluate each GTRT tree based on fitness function presented in Eq 29;

23:    **BS:** Determine the best grasshopper and set it as $\hat{T_{d}}$;

24:   **end while**

25:    **BS:** Extract the best GTRT from $\hat{T_{d}}$;

26:  **end if**

27:  *t*_*counter*_ = *t*_*counter*_ + 1;

28: **end while**

  **End**

## 6 Simulation and results

In order to analyze the performance of CTTRG, this method is run on NS2, and the experimental results of CTTRG are compared to those of TBSEER [21], TESRP [22] and TSSRM [23]. The reasons behind the selection of these methods are summarized below:

- CTTRG, TBSEER, TSSRM, and TESRP are energy-efficient methods and pay attention to the energy parameter in the routing process. In addition, CTTRG, TBSEER, and TSSRM have considered an energy parameter in the trust evaluation process.
- CTTRG, TBSEER, TSSRM, and TESRP have presented powerful and distributed trust mechanisms in their methods.
- CTTRG, TBSEER, TSSRM, and TESRP have the ability to detect and isolate hostile nodes in the network. As a result, a secure environment is provided for data transfer between trusted nodes.
- TBSEER, CTTRG, and TSSRM can resist various attacks such as Wormhole, Gray hole, and Flooding.
- TBSEER and CTTRG are hierarchical methods and use the clustering technique, which will improve the energy efficiency of these methods.

In the simulation operation, various methods deal with five attacks, namely BH, SH, WH, GH, and FA, and their results are evaluated and analyzed. In this operation, there are 100 sensor nodes in a network with size 100×100*m*^2^. Each node has energy equal to one joule, and its initial trust level is 0.5. Moreover, the trust threshold is 0.35 so, if the trust of the nodes is lower than this threshold, those nodes are marked as hostile nodes. Moreover, the sizes of control packets and data packets are 400 bits and 4, 000 bits, respectively. Table 2 states the most important simulation settings.

**Table 2:** Simulation settings.

Scale	Value
Simulation software	NS2
Compared schemes	TBSEER, TSSRM, and TESRP
Routing attacks	BH, SH, WH, GH, and FA
Network dimensions	100×100*m*^2^
The number of nodes	100
Maximum energy of nodes	1 J
Primary trust of nodes	0.5 J
Trust threshold	0.35
Control packet size	400 bits
Data packet size	4000 bits

### 6.1 Trust evaluation

**Fig 3:**
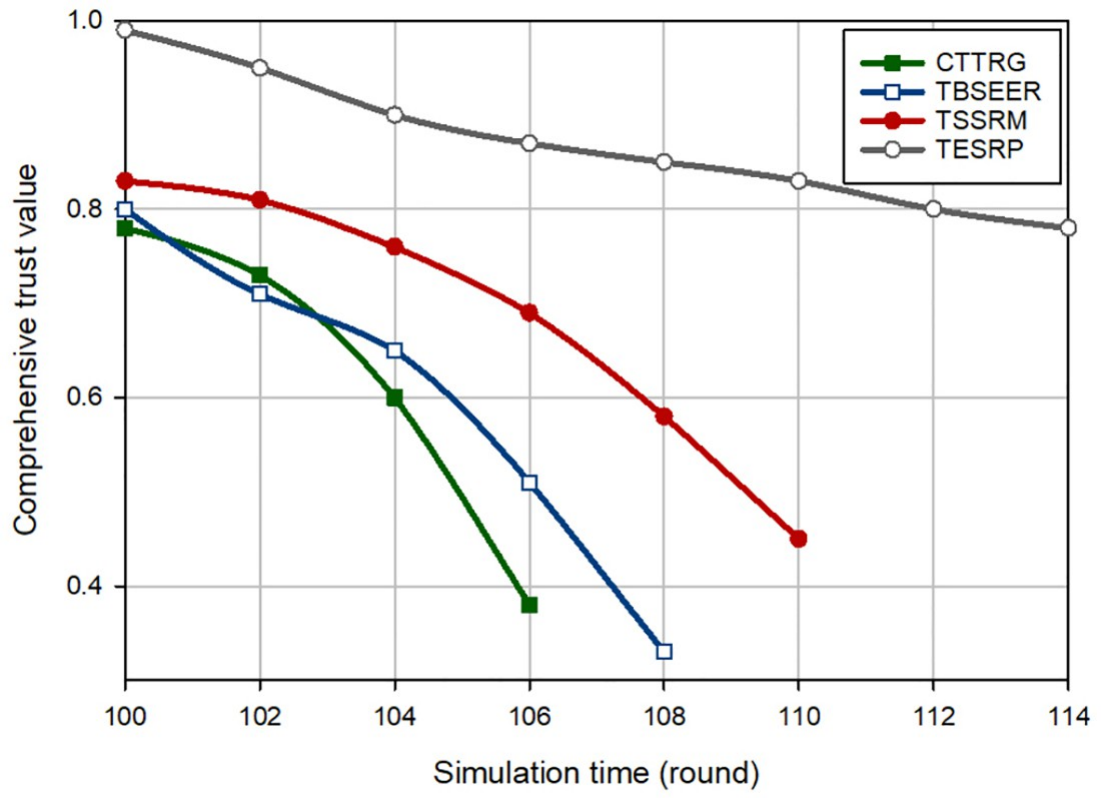
Comparison of the trust changes of BH nodes in different schemes.

**Fig 4:**
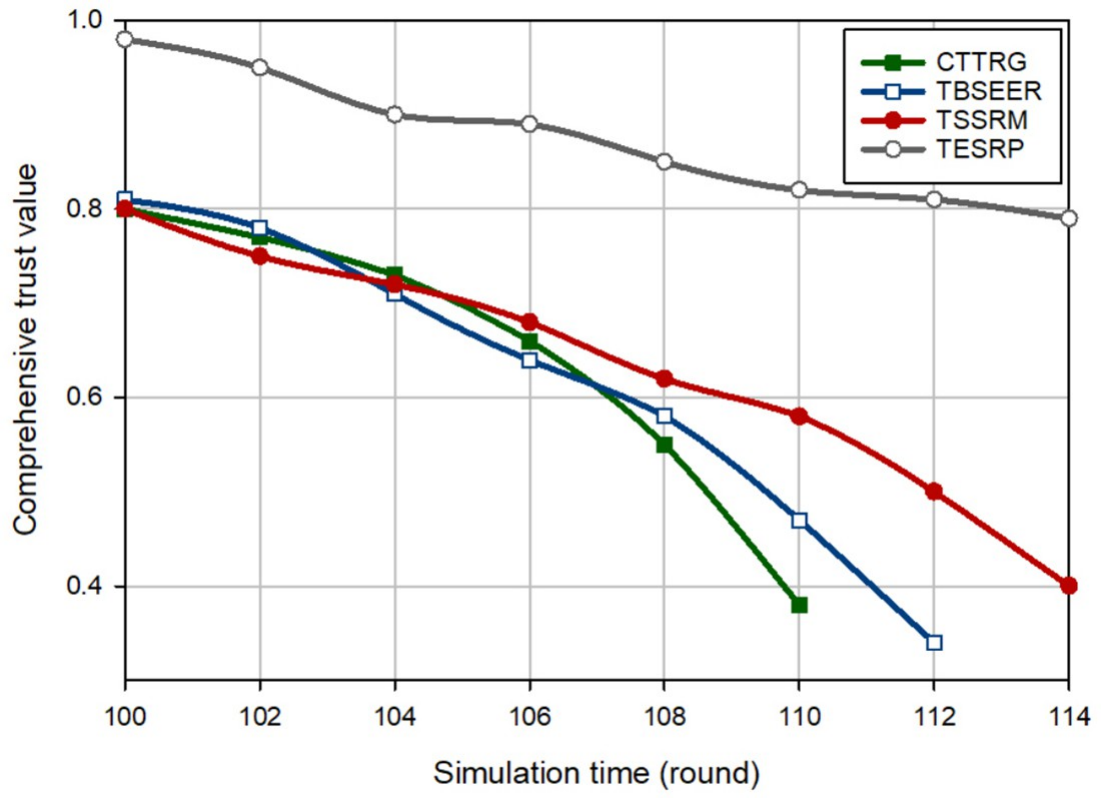
Comparison of the trust changes of GH nodes in different schemes.

**Fig 5:**
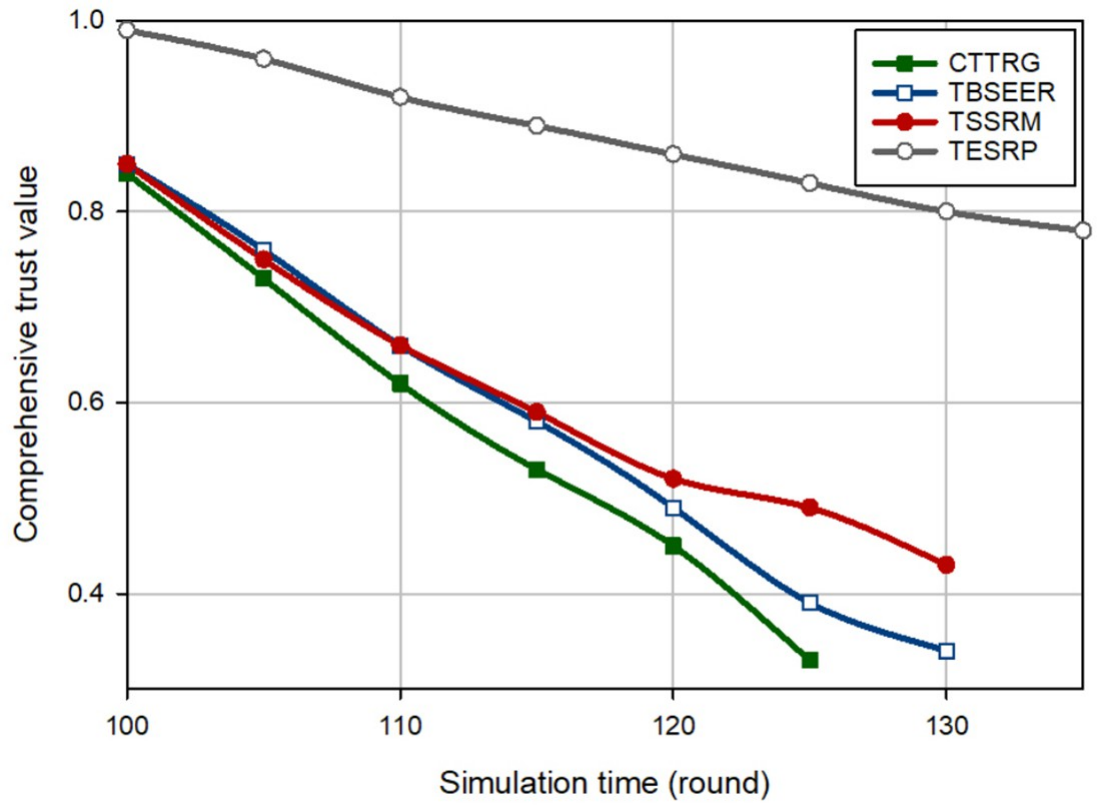
Comparison of the trust changes of SH nodes in different schemes.

**Fig 6:**
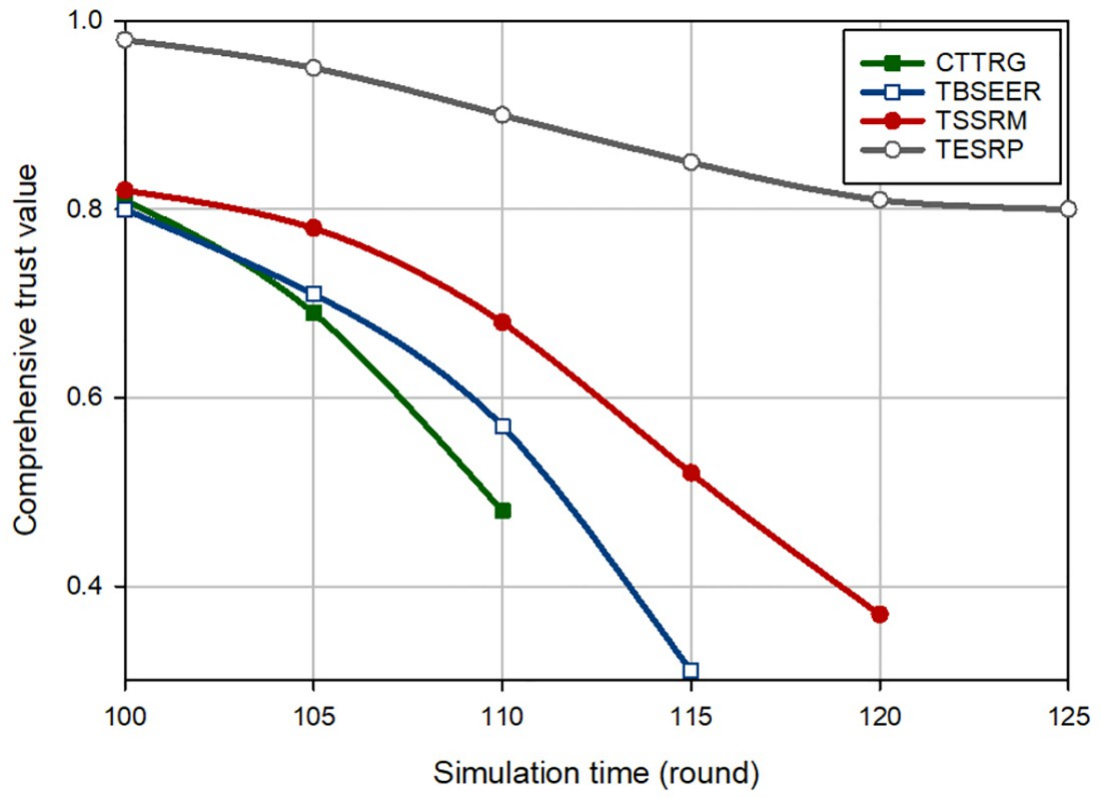
Trust changes of FA nodes in different schemes.

**Fig 7:**
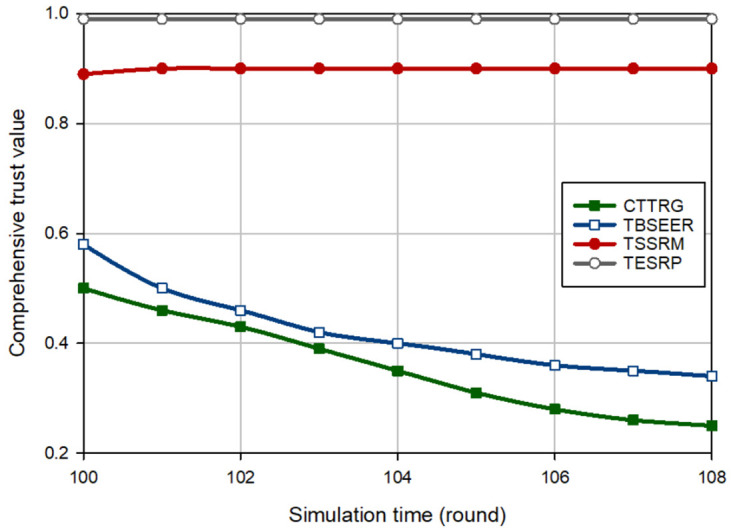
Trust changes of WH nodes in different schemes.

Fig 3 displays an evaluation of the trust of the hostile nodes (i.e. BH nodes) for different methods. In this experiment, it is assumed that in round 100, five BH nodes are injected into the network. According to Fig 3, CTTRG identifies these nodes quickly and only after seven rounds. This proves the powerfulness of the TVT model presented in CTTRG for detecting BH nodes. Among other routing schemes, TBSEER also works well so that the BH nodes have been identified and removed after 8 rounds. However, TESRP shows the weakest performance in identifying BH nodes. In Fig 4, it is assumed that five GH nodes are entered into the network in round 100. Note that it is more difficult to diagnose this attack compared with the BH attack because GH nodes are smarter and focus only on a particular type of packets and behave normally in other cases. This is well visible in Fig 4 because CTTRG detects these nodes at a slower speed and requires 11 rounds to isolate these nodes in the network. While TBSEER can recognize and separate GH nodes in 12 rounds. It has a good performance. In Fig 5, a SH attack occurs on the network. The detection speed of CTTRG and TBSEER is slow and close to TSSRM, so that CTTRG identifies the SH nodes after 25 rounds. However, TBSEER requires 28 rounds to detect and separate these SH nodes. The reason for the successful performance of the suggested scheme in this experiment is that the TVT system used in CTTRG contains a dynamic and time-variant coefficient. It will be reduced or increased using the historical trust values of each node. Hence, CTTRG quickly reduces the trust level of hostile nodes in each round and identifies these nodes in shorter rounds. In Fig 6, a FA attack occurs on the network, and 5% of the network nodes are hostile. All routing approaches slowly reduce the trust level of the FA nodes and detect such an attack. However, our scheme has shown the best performance in identifying this attack because CTTRG uses a parameter called the FA probability to calculate the trust value. Furthermore, it quickly detects these nodes based on the energy level change and the number of duplicate packets. Finally, in the last experiment, Fig 7 considers the WH nodes in the network. CTTRG has identified the attack in 5 rounds and TBSEER has identified the WH nodes in 8 rounds. However, TSSRM and TESRP cannot detect this attack because they do not diminish the trust level of the WH nodes.

### 6.2 Detection speed

**Fig 8:**
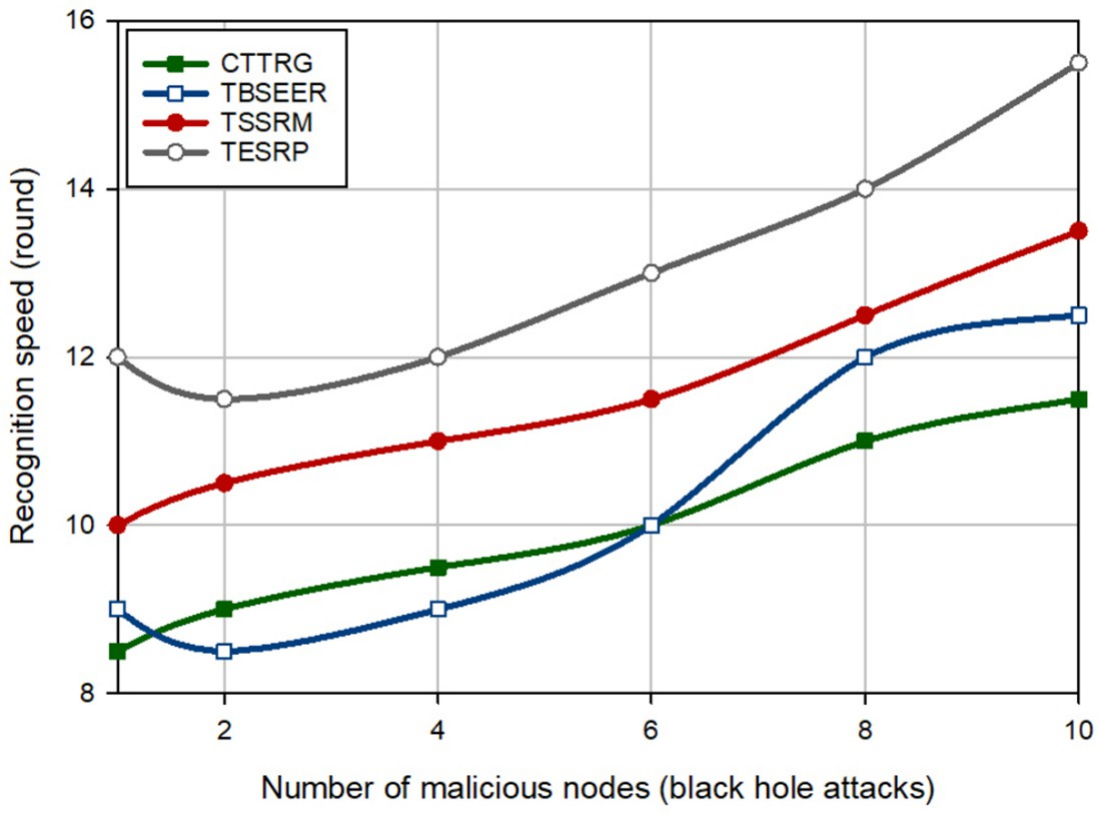
Comparison of the detection speed of different methods in a BH attack.

**Fig 9:**
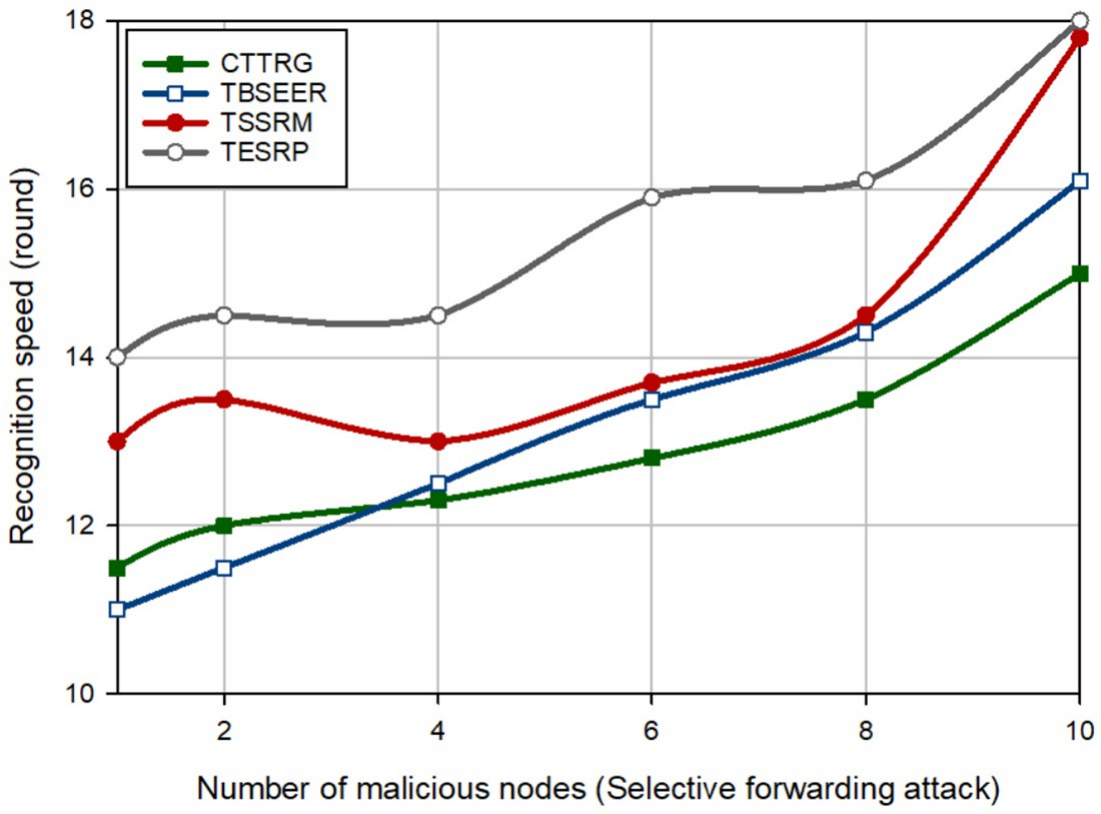
Comparison of the detection speed of different methods in a GH attack.

**Fig 10:**
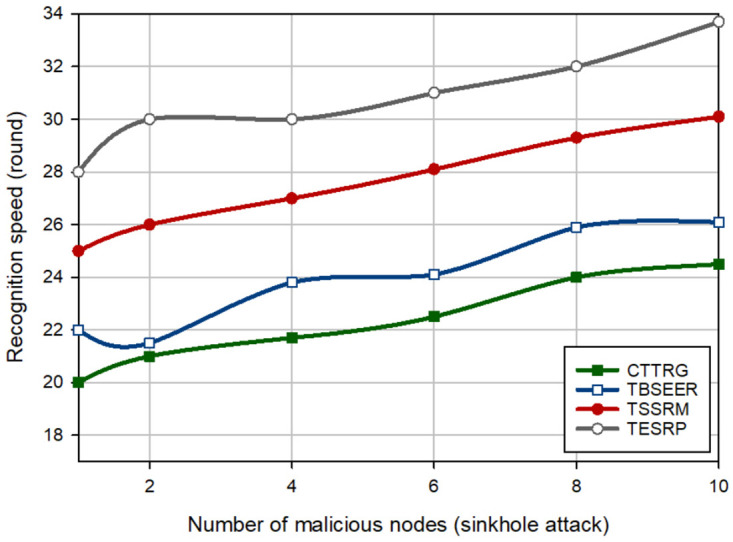
Comparison of the detection speed of different methods in SH attack.

**Fig 11:**
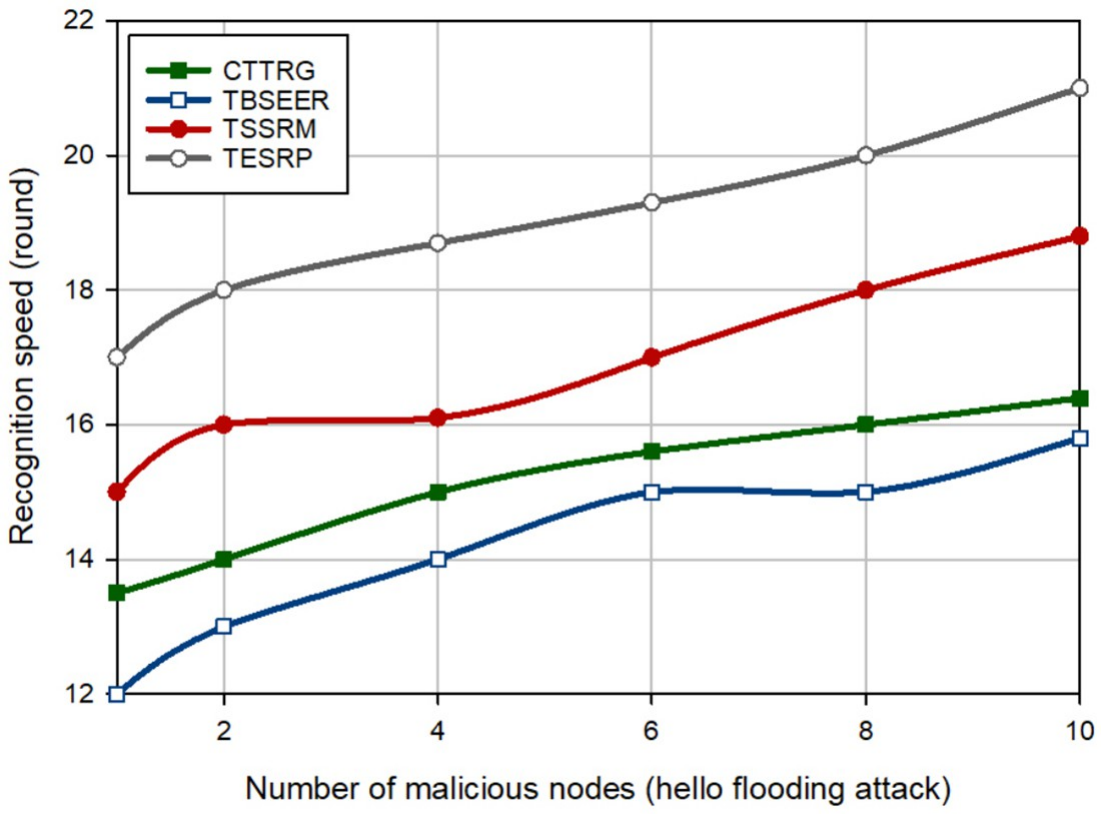
Comparison of the detection speed of different methods in FA attack.

In the next experiment, the detection speed of different schemes is evaluated in the presence of several hostile nodes (between 1–10). Fig 8 shows the detection speed of different schemes for a BH attack. This figure proves that CTTRG has the best detection speed and diagnoses the BH nodes approximately 2.46%, 13.74%, and 23.69% faster than TBSEER, TSSRM, and TESRP, respectively. Moreover, Fig 9 compares the detection speed of different approaches for a GH attack. According to this figure, CTTRG has improved the detection speed of GH nodes by 2.28%, 9.82%, and 17.097% compared to TBSEER, TSSRM, and TESRP, respectively. In addition, Fig 10 evaluates different schemes in terms of the detection speed of SH attacks. In this figure, CTTRG increases the detection speed of the SH nodes by 6.78%, 19.22%, and 27.62% in comparison with TBSEER, TSSRM, and TESRP, respectively. Finally, Fig 11 shows the performance of various schemes for identifying FA nodes. According to this figure, CTTRG has a lower speed (approximately 5.45%) than BSEER to diagnose FA nodes. However, our scheme is 10.34% and 20.63% faster than TSSRM and TESRP, respectively. Obviously, an opposite relationship is between the number of hostile nodes and the detection speed so that if a lot of hostile nodes attack the network, the detection rate will be lowered in different schemes because the nodes can participate with each other, and this decreases the accuracy of the recommendations provided by the recommended nodes. However, in CTTRG, these recommendations are prioritized, meaning that if a recommender node is not reliable or secure, its recommendation is very different from the direct trust evaluated by the trusted nodes and hence, this recommendation has less priority than other recommendations, and has little effect on the final trust.

### 6.3 Packet loss rate (PLR)

**Fig 12:**
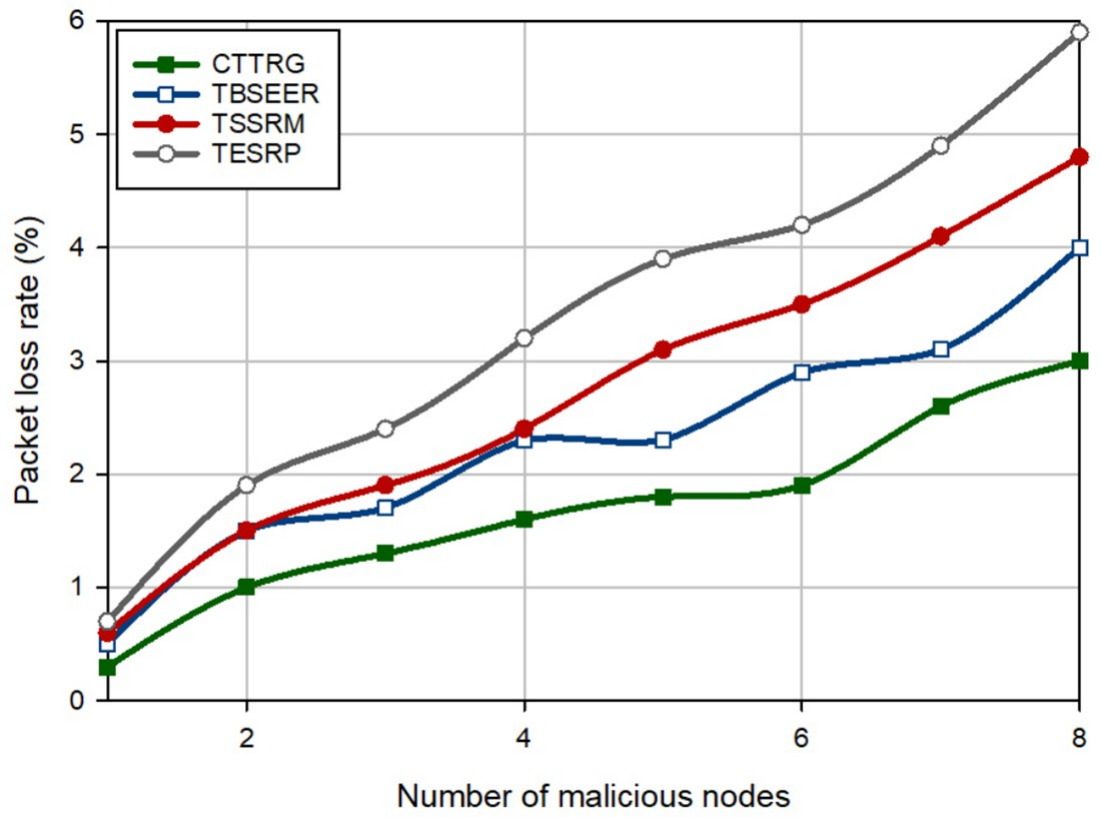
Comparison of PLR in different schemes.

PLR means the ratio of the number of packets, which do not arrive at the BS to all packets sent to BS. The PLR results in different schemes are stated in Fig 12 when there are 1–8 hostile nodes in the network. According to this figure, CTTRG has the lowest PLR and reduces it by 26.23%, 38.36%, and 50.18% in comparison with TBSEER, TSSRM, and TESRP, respectively. This is due to the powerful security mechanism designed in CTTRG, which identifies hostile nodes quickly. It will also prevent the effect of malicious nodes and reduces the number of missing data packets. On the other hand, three factors, namely the distance between each CH to its parent, the trust level of the network nodes, and their energy are considered when forming a GTRT tree. This causes the creation of a stable and secure tree between CHs.

### 6.4 Delay

**Fig 13:**
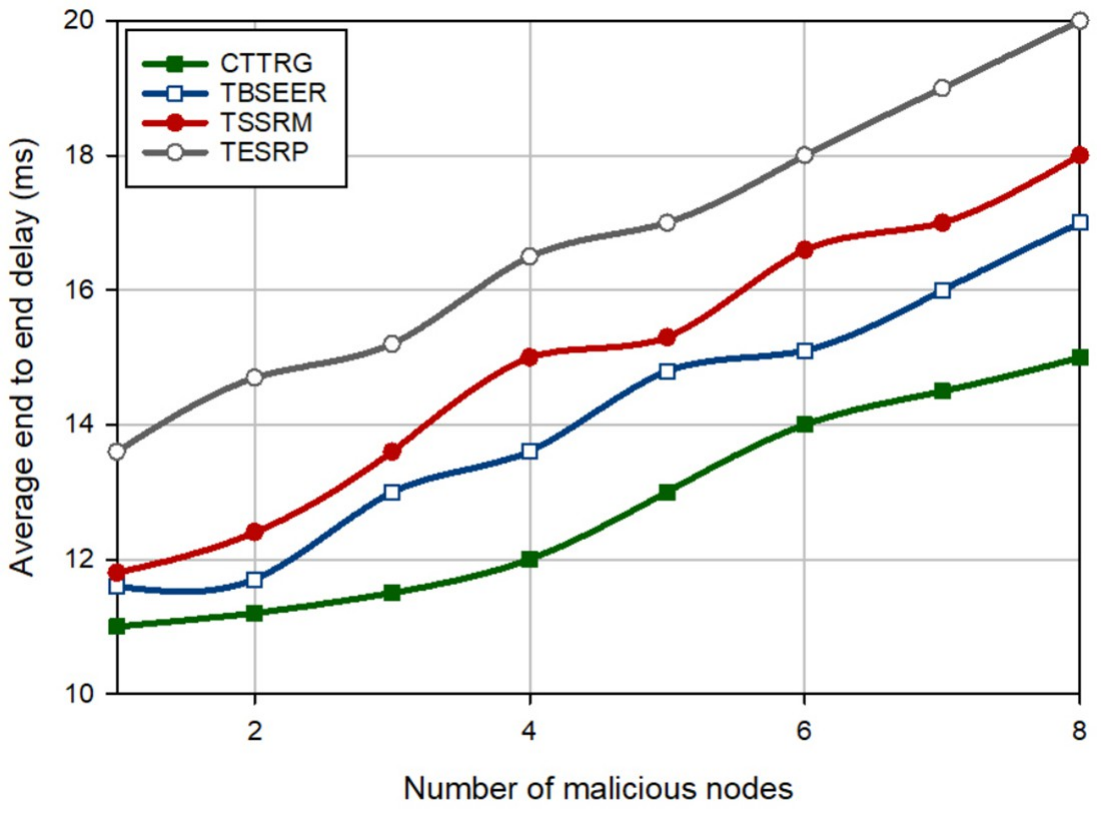
Comparison of delay in different methods.

Delay is the average time required to send a packet from the source node to the BS. In Fig 13, CTTRG decreases delay by 9.40%, 14.62%, and 23.73% compared to TBSEER, TSSRM, and TESRP, respectively. In CTTRG, delay is reduced in the routing process because it transfers data packets through the optimized GTRT tree. As shown in Fig 13, delay is directly proportional to the number of hostile nodes in the network, so if the number of these nodes is high, the data transfer operation is delayed in all routing methods. This is because the security systems can difficultly detect a high number of malicious nodes on the network and hence, some hostile nodes are not identified and will have a negative effect on network performance.

### 6.5 Energy consumption

**Fig 14:**
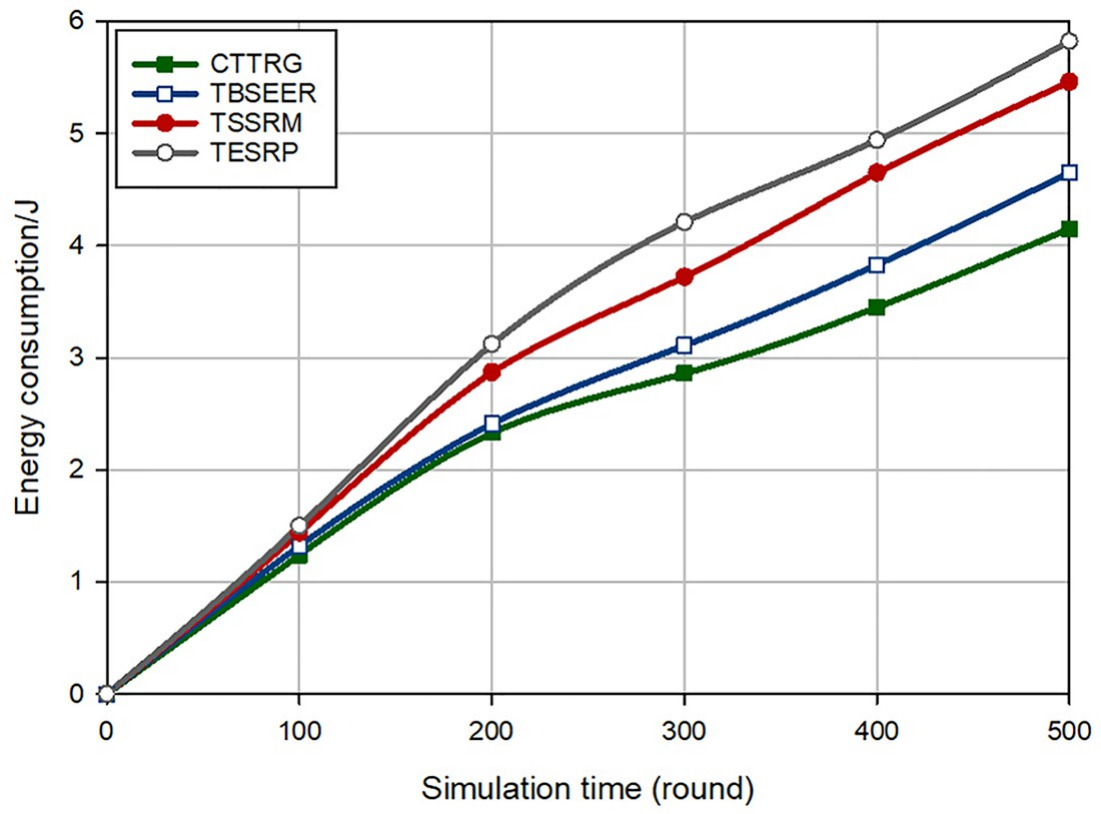
Comparison of energy consumption in different methods.

In Fig 14, the energy consumed in different schemes is compared with each other. Based on this figure, it can be seen that CTTRG has improved energy consumption by 8.42%, 22.66%, and 28.38% compared to TBSEER, TSSRM, and TESRP, respectively. The main reason for this improvement is that CTTRG uses tree-cluster topology, which greatly increases the efficiency of our method in terms of energy consumption. On the other hand, in the GTRT tree construction process, the remaining energy of cluster heads is considered as an important factor in the fitness function. As a result, the designed GTRT tree balances the energy consumption of CHs in the network and increases network lifetime.

## 7 Conclusion

In this paper, a cluster-tree-based trusted routing method using the grasshopper optimization algorithm is proposed for WSNs. CTTRG contains two components: the time-variant trust mechanism and the GOA-based trusted routing tree. The TVT mechanism analyzes the behavior of sensor nodes and measures their trust level based on the three criteria, including the BH, GH, and SH probability, the WH probability, and the FA probability. Additionally, the GTRT tree is looking for safe and trust communication paths between CHs and BS. CTTRG is run on NS2 and its performance is compared with TBSEER, TSSRM, and TESRP. The experimental results show that CTTRG lowers the detection speed of BH nodes by 2.46%, 13.74%, and 23.69%, the detection speed of GH nodes by 2.28%, 9.82%, and 17.097%, and the detection speed of SH nodes by 6.78%, 19.22%, and 27.62% in comparison with TBSEER, TSSRM, and TESRP, respectively. However, CTTRG has a lower speed (approximately 5.45%) than TBSEER to diagnose FA nodes. In addition, our scheme lowers PLR by 26.23%, 38.36%, and 50.18% and delay by 9.40%, 14.62%, and 23.73% compared to TBSEER, TSSRM, and TESRP, respectively. In future research directions, we will use new techniques, for example, machine learning or meta-heuristic algorithms to enhance the strength of the trust system in CTTRG. Furthermore, GTRT tree can be constructed using different nature-based algorithms to obtain the best tree.
